# *Lactobacillus gasseri* SBT2055 Induces TGF-β Expression in Dendritic Cells and Activates TLR2 Signal to Produce IgA in the Small Intestine

**DOI:** 10.1371/journal.pone.0105370

**Published:** 2014-08-21

**Authors:** Fumihiko Sakai, Tomohiro Hosoya, Aiko Ono-Ohmachi, Ken Ukibe, Akihiro Ogawa, Tomohiro Moriya, Yukio Kadooka, Takuya Shiozaki, Hisako Nakagawa, Yosuke Nakayama, Tadaaki Miyazaki

**Affiliations:** 1 Milk Science Research Institute, Megmilk Snow Brand Co. Ltd., Minamidai, Kawagoe, Saitama, Japan; 2 Department of Probiotics Immunology, Institute for Genetic Medicine, Hokkaido University, Kita-ku, Sapporo, Japan; Massachusetts General Hospital, United States of America

## Abstract

Probiotic bacteria provide benefits in enhancing host immune responses and protecting against infection. Induction of IgA production by oral administration of probiotic bacteria in the intestine has been considered to be one reason for this beneficial effect, but the mechanisms of the effect are poorly understood. *Lactobacillus gasseri* SBT2055 (LG2055) is a probiotic bacterium with properties such as bile tolerance, ability to improve the intestinal environment, and it has preventive effects related to abdominal adiposity. In this study, we have found that oral administration of LG2055 induced IgA production and increased the rate of IgA^+^ cell population in Peyer's patch and in the lamina propria of the mouse small intestine. The LG2055 markedly increased the amount of IgA in a co-culture of B cells and bone marrow derived dendritic cells (BMDC), and TLR2 signal is critical for it. In addition, it is demonstrated that LG2055 stimulates BMDC to promote the production of TGF-β, BAFF, IL-6, and IL-10, all critical for IgA production from B cells. Combined stimulation of B cells with BAFF and LG2055 enhanced the induction of IgA production. Further, TGF-β signal was shown to be critical for LG2055-induced IgA production in the B cell and BMDC co-culture system, but TGF-β did not induce IgA production in a culture of only B cells stimulated with LG2055. Furthermore, TGF-β was critical for the production of BAFF, IL-6, IL-10, and TGF-β itself from LG2055-stimulated BMDC. These results demonstrate that TGF-β was produced by BMDC stimulated with LG2055 and it has an autocrine/paracrine function essential for BMDC to induce the production of BAFF, IL-6, and IL-10.

## Introduction

Probiotics are live microorganisms which when they are administered in adequate amounts confer health benefits to the host [1]. Probiotic bacteria, mainly belonging to the class of lactic acid bacteria (LAB), are well known to induce beneficial effects in human and animal health. In particular, lactobacilli are characterized by the production of lactic acid and are commonly applied to many vegetable, meat, and dairy fermentations. These bacteria can influence the composition and activity of the gut microbiota. Currently, there is a general consensus that orally administered probiotic bacteria contribute to immune homeostasis by altering the microbial balance or by interacting with the host immune system [2]–[4]. In particular, the interplay between the mucosa-associated immune system and microbiota certainly plays a pivotal role in mucosal tissue homeostasis as well as in protection against infectious and inflammatory diseases occurring at mucosal sites [5].

In the intestinal tract, IgA is the most abundant immunoglobulin isotype, with up to 3 g of secretory IgA secreted into the human intestinal lumen per day [6], [7]. The IgA plays an important role in the host defense against mucosally transmitted pathogens, preventing commensal bacteria from binding to epithelial cells, and neutralizing their toxins to maintain homeostasis at the mucosal surfaces [8]. These functions are beneficial for the host as they reduce the risk of infection and maintain an intestinal environment accommodating to the appropriate commensal population. In humans, individuals with IgA deficiency have increased rates of respiratory and gastrointestinal infectious diseases, and lympho-proliferative disorders of the small intestine [9]. It has been reported that intestinal commensal bacteria induce IgA production by developing gut associated lymphoid tissue (GALT) in the small and large intestine [10]–[13]. Within the network of intestinal immunity, dendritic cells (DCs) play a critical role in the switching between stimulating immune regulation or activating immune responses of commensal microbiota [14]. It has been reported that administration of some strains of lactobacilli or bifidobateria increase the mucosal IgA production [15]–[19]. However, the mechanism of the induction of IgA production by probiotic bacteria has not been established in detail.

The *L. gasseri* strain SBT2055 (LG2055) is a human intestine-originating probiotic bacterium with properties including bile tolerance [20], the ability to become established in the intestine, and lowered both faecal bacterial population of *Staphylococcus* and faecal concentration of p-cresol. [21], [22], having a cholesterol lowering effect in humans with mild hepercholesterolemia [23], and preventing abdominal adiposity in rats [24], [25] and humans [26], among others. A further recent finding regarding LG2055 has reported that LG2055-fed mouse dams reduced rotavirus infections in pups and elevated RV-specific IgA levels in breast milk originating from the stomach [27]. This finding raises the possibility that administration of LG2055 may induce IgA production in the intestinal tract, where IgA is most abundantly produced in the tissue.

In the present study, we examined whether the administration of LG2055 to mice augmented IgA levels in the intestine, and also the precise molecular mechanisms for the IgA induction by an *in vitro* culture system using bone marrow derived dendritic cells.

## Materials and Methods

### Mice

Male SPF BALB/c mice were purchased from Charles River Japan (Yokohama, Japan) or SLC Inc. (Shizuoka, Japan) and maintained at the animal experimental facilities of Megmilk Snow brand Co., Ltd. or Hokkaido University. The mice were given free access to food and distilled water. All procedures for animal care and use complied with the animal experimentation regulations of the Milk Science Research Institute of Megmilk Snow Brand Co., which is based on the guidelines proposed by the Science Council of Japan, and the guidelines of the Bioscience Committee of Hokkaido University. All animal experiments were approved by the Animal Care and Use Committee of Milk Science Research Institute of Megmilk Snow Brand Co. and the Animal Care and Use Committee of Hokkaido University.

### Preparation and growth condition for *Lactobacillus gasseri* SBT2055 (LG2055)

LG2055 is a bacterial strain derived from a fecal specimen of a healthy adult, which had been isolated by Fujiwara et al. [21] and deposited in the International Patent Organism Depository, National Institute of Advanced Industrial Science and Technology (Tsukuba, Ibaraki 305-8566, Japan). LG2055 was cultivated at 37°C in 2 liters of MRS broth (Difco, Detroit, Mich.) for 18 hours and harvested by centrifugation at 5,000×g for 10 minutes at 4°C. To prepare the LG2055 cells powder for *in vivo* studies, the harvested cells were washed twice with sterile distilled water and suspended in 500 mL of 10% (w/v) lactose solution. Suspension of LG2055 was lyophilized to make powder. The lyophilized powder consist of 1.2×10^11^ cfu/g viable LG2055 and 65% lactose. LG2055-lactose powder was stored at −80°C for experiments. To prepare the LG2055 cells for *in vitro* studies, cells washed with distilled water were suspended in distilled water, and lyophilized to make powder. LG2055 powder was resuspended in PBS and heated at 80°C for 30 min. The ability of other strains of *Lactobucillus* spp., which were *L. gasseri* JCM1131^t^ (LG1131T), *L. helveticus* SBT2171 (LH2171), *L. acidophilus* SBT2062 (LA2062), to induce the IgA and cytokines production in an *in vitro* culture system were examined for comparison with that of LG2055. LH2171 is used for manufacturing of natural cheeses [28] and LA2062 is a probiotic bacterium utilized for manufacturing of fermented milk products in past times [29]. The cells of these three strains were prepared by the same methods used to prepare the LG2055 cells for *in vitro* studies.

### Orally administration of LG2055 to mice

Six-week-old male Balb/c mice were used in this study. After a one-week adaptation period, the mice were separated into two groups: the control group (n = 10), and the LG2055-treated group (n = 10). Each group mouse had a similar mean body weight. Mice were given an experimental diet prepared according to AIN93 formation supplemented with 1% LG2055-lactose powder for LG2055 group or AIN93 supplemented with 0.65% lactose for control group to equalize lactose contents in both diets. Experimental diets were replaced on a daily basis by fresh diets. Stocks of experimental diets were stored at −20°C and the viable cell number of LG2055 in the diet was confirmed as more than 1.0×10^9^ cfu/g throughout the experiments. All mice were fed ad libitum experimental diets and distilled water for 5 weeks.

### Extraction of intestinal tissues

After the mice were sacrificed under isoflurane anesthesia, the small intestine and colon were removed carefully from mice of control or LG2055-treated group. Small intestinal lavage fluid was collected by washing out with 10 ml of ice-cold PBS for determination of IgA level. Peyer's patches were excised from the small intestine for FACS analysis. The colon was isolated, open longitudinally to remove the intestinal contents, and then washed with PBS. The tissue samples from jejunum, ileum, and colon were weighted and added a 40-fold volume of PBS containing protease inhibitor cocktail (Roche, Schweiz) in 50 mM Tris-HCl (pH 6.8) buffer. These tissues were homogenized on ice using a ULTRA-TURRAX T25 (IKA-Werke GmbH & CO., Germany). The suspension was centrifuged at 10,000×g for 15 min, and the supernatant was used for the detection of intestinal IgA levels by mouse IgA ELISA (Bethyl Laboratories, Inc., TX) and total protein levels by BCA Protein Assay kits (PIERCE, IL).

### Fecal sample preparation

The fecal samples collected were lyophilized and suspended in PBS containing 50 mM EDTA and 0.1 mg/ml soybean trypsin inhibitor. Fecal suspension was vigorously vortexed and centrifuged at 15,000×g for 10 min at 4°C. The supernatants were diluted appropriately for mouse IgA ELISA analysis.

### Preparation of Peyer's patch cells and lamina propria cells from mice after oral administration of LG2055

Peyer's patches (PP) cells and lamina propria (LP) cells from small intestine were prepared for analysis of IgA^+^ cells by FACS. PP dissected from the small intestine were mechanically disrupted in the cell culture medium (RPMI-1640 containing 10% FBS, 10 mM HEPES buffer, 2 mM l-glutamine, 100 U/mL of penicillin, 100 µg/mL streptomycin, and 0.05 mM 2-mercaptoethanol). PP cells were washed with PBS and resuspended in the cell culture medium. For isolation of LP cells, the small intestines were removed after excluding PP, and then cut into 5–7 pieces. The small intestines were washed three times with 40 mL of Hanks' balanced salt solution (HBSS) (sigma, MO) supplemented with 5% FBS and 5 mM EDTA in a 50 mL tube and incubated at 37°C for 30 min with shaking at 150 rpm. The tissues were cut into smaller pieces, and were incubated with RPMI1640 supplemented with 1 mg/ml collagenase (Sigma) and 10 µU/ml DNase I (Roche) at 37°C for 60 min with stirring. Collected cells were placed on the boundary between 44% and 70% concentration of Percoll solution (GE Healthcare, UK), and were centrifuged at 1500×g for 20 min. These cells were washed and used as LP cells.

### Flow cytometric analysis

PP cells and LP cells were pretreated with FcR blocking reagent (Miltenyi Biotec) and then stained with the following mAbs: FITC-labeled anti-mouse IgA (C10-3, BD Bioscience, NJ), PE-labeled anti-mouse B220 (RA3-6B2, eBioscience, CA). Live cells were gated based on 7AAD exclusion during acquisition on a FACS CantII (BD Bioscience). Flow cytometric analysis was performed using FACS Diva software (BD Bioscience).

### Depletion or isolation of CD11c^+^ cells from PP cells, and naïve B cell isolation from spleen cells

Six to ten-week-old male Balb/c mice were euthanized by overdose of inhalant anesthetic and sacrificed. PPs collected from small intestines were shaken for 45 min at 37°C in PBS containing 2 mM EDTA to remove epithelial cells and subsequently treated with 2 mg/ml collagenase D (Roche) and 10 µg/ml DNaseI (Roche) in complete RPMI1640 at 37°C for 30 min. Digested PPs were harvested and passed through a 70 µm cell strainer (BD biosciences). Single cells were labeled with microbeads coated with anti-CD11c antibody (Miltenyi Biotec) according to the manufacturer's instruction. For depletion of CD11c^+^ cells, labeled PP cells were applied to LD column (Miltenyi Biotec) and passing through unlabeled cells were collected, used as CD11c depleted cells. For isolation of CD11c^+^ cells, labeled PP cells were applied to LS column (Miltenyi Biotec) and positively selected cells were collected. To obtain the CD11c^+^ cells further purified to greater than 95%, magnetic-isolated cells were labeled with APC-conjugated anti-mouse CD11c (N418, BioLegend), and PE-conjugated anti-mouse CD19 (BD biosciences) antibodies for depletion of the contaminating B cells. CD11c^+^CD19^-^ cells were collected by sorting using a FACSAria (BD biosciences), and used as CD11c^+^ cells derived from PP cells (purity >95%, CD11c^+^). To prepare B cells, spleen cells from naïve BALB/c mice were separated by anti-mouse IgM coated MicroBeads (Miltenyi Biotec). Positively selected IgM^+^ cells were used as B cells (purity >95%, IgM^+^).

### Generation of bone marrow derived dendritic cell (BMDC)

Six to ten-week-old male Balb/c mice were euthanized by overdose of inhalant anesthetic and sacrificed. The femurs and tibias were removed, cleaned, and sterilized. The bone marrow was flushed from bones by use of a syringe containing culture medium. For BMDC isolation, the bone marrow cells were washed and cultured at 4.0×10^6^ cells/dish (10 cm culture dish) in culture medium supplemented with 40 ng/ml GM-CSF (Wako, Japan) for 8 days. Harvested cells were blocked with anti-mouse Fc receptor (BioLegend) and then stained with FITC-labeled anti-CD11c (N418, BioLegend) and PE-labeld anti-mouse MHC-II (I-A/I-E; M5, BioLegend) antibodies. CD11c^+^ MHC-II^+^ cells were sorted by BD FACSAria II (BD Biosciences, CA) and used as BMDC (purity >95%, CD11c^+^ MHC-II^+^ cells). For analysis of the function of LG2055 against BMDC, BMDC (5.0×10^5^ cells/mL) were cultured with or without LG2055 (20 µg/mL) in presence or absence of SB505124 (5 µM, Sigma) in 12 well plate (BD Bioscience) for 48 h. After incubation, culture supernatants were collected for measurement of cytokines and total RNA were prepared from BMDC for real time-PCR analysis.

### Assay for IgA amounts by *in vitro* cell culture system

To evaluate the mechanism for IgA production *in vitro*, B cells (5×10^5^) were either directly stimulated with LG2055 (10 µg/ml), BAFF (500 ng/ml, Sigma), TGF-β1 (0.001–1 ng/ml, R&D Systems), and LPS (10 µg/mL, InvivoGen) or co-cultured with CD11C^+^ cells derived from PP cells (0.5×10^5^) or BMDC (1×10^5^) in the presence or absence of LG2055 (10 µg/ml), LE135 (500 nM), SB505124 (5 µM), anti-human/mouse TLR2 (5 µg/ml), Pam3CSK4 (1 µg/mL, InvivoGen, CA), and FSL-1 (1 µg/mL, InvivoGen) in 96 well-round bottom plate (BD Bioscience) for 7 days. To block cell-to-cell contact, B cells (2×10^6^) and BMDC (4×10^5^) were cultured in 24 well culture plate (BD Bioscience) with or without a transwell device (BD Bioscience) in the presence or absence of LG2055 (10 µg/ml) for 7 days. The level of IgA in the culture supernatants was measured by mouse IgA ELISA.

### RNA extraction and quantitative real time-PCR analysis

Total RNA from BMDC was extracted with TRIzol reagent (Molecular Research Center, Inc.) and reverse transcribed by the TaqMan Reverse Transcription Reagent kit (Applied Biosystems) according to the manufacturer's instructions. The following TaqMan Gene Expression Assay were purchased (Applied Biosystems): BAFF (Assay ID Mm00446347_m1), APRIL (Assay ID Mm00840215_g1), RALDH2 (Assay ID Mm00501306_m1), β-actin (Assay ID Mm00607939_s1). Amplifications were carried out in a total volume of 20 µL containing 1x TaqMan Universal PCR Master Mix (Applied Biosystems). The cycling parameters were initiated by 20 s at 95°C, followed by 40 cycles of 3 s at 95°C and 30 s at 60°C using the ABI Prism 7000 (Applied Biosystems). The amplifications were normalized by the expression of β-actin encoding gene.

### Quantification of antibody isotypes and cytokine by ELISA

Tissue extract, intestinal gavage, serum, and culture supernatants were analyzed for the amount of total IgA or IgG antibody by mouse IgA or IgG ELISA (Bethyl Laboratories, Inc., TX). Mouse IL-5, IL-6, IL-10, latent TGF-β (BioLegend), and BAFF (R&D systems Inc.) in cell culture supernatants were analyzed using commercial available ELISA kits according to the manufacturer's instructions.

### Statistical analysis

Data were expressed as means ± standard deviations. Level of significance was determined by one-way ANOVA and Tukey-Kramer post test in experiment for comparison of IgA induction of non-treated LG2055 with that of heat-treated LG2055, one-way ANOVA and Dunnett's post test in examinations of IgA induction by four strains of *Lactobacillus* spp., and Student *t* test in the other experiments and *p* values<0.05 were considered to be statistically significant.

## Results

### LG2055 induces the production of IgA in the small intestine

**Figure 1:**
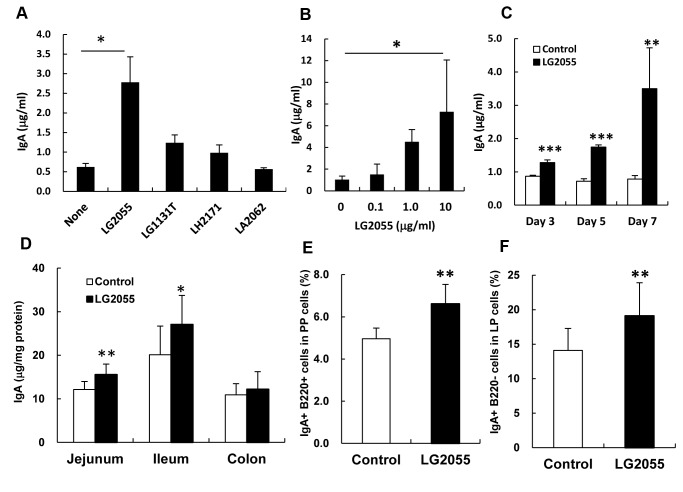
Augmentation of IgA production by LG2055 *in vitro* and *in vivo*. Whole PP cells were cultured with or without each of the four *Lactobacillus* strains (LG2055, *L. gasseri* JCM1131^t^ (LG1131T), *L. helveticus* SBT2171 (LH2171), *L. acidophilus* SBT2062 (LA2062), 10 µg/ml) for 7 days (A). Whole PP cells were cultured with 0, 0.1, 1.0, and 10 µg/ml of LG2055 for 7 days (B). Whole PP cells were cultured with or without LG2055 (10 µg/ml) for 3, 5, and 7 days (C) The amounts of IgA in culture supernatants were determined by ELISA. Each experiment was done with tripricate cultures; data are shown as the mean ± SD. The values for cells cultured with lactic acid bacteria are compared with that of without the bacteria by one-way ANOVA, Dunnett's post test (A and B) and the *t*-test (C). Significant differences are indicated by * P<0.05, ** P<0.01, *** P<0.001. LG2055 was orally administrated to BALB/c mice for 5 weeks. Amounts of total IgA in intestinal tissue extracts (D) were determined by ELISA. The population of IgA^+^ B220^+^ cells in PP cells (E) and IgA^+^ B220^-^ cells in LP cells (F) was analyzed by FACS. Representative data from two independent experiments are shown. Data are shown as the mean ± SD (number of mice n = 10). Significant difference from control group at *P<0.05, **P<0.01 was shown by the *t*-test (D, E, and F).

To identify the *Lactobacillus* strain to induce IgA production, we assessed its induction in PP cells cultured with or without four strains of *Lactobacillus* species. LG2055 exhibited a marked induction of IgA production in comparison to the other strains (Figure 1A). Induction of IgA production by LG2055 increased in a time- and dose-dependent manner (Figure 1B, C). Next, we evaluated the IgA induction in the intestine of mice by the oral-administration of LG2055. Mice were given the control diet or the diet supplemented with LG2055 for 5 weeks. Throughout the experiments, the food intake and body weight of the control and LG2055 diet groups did not differ (data not shown). The amount of total IgA in the intestinal tissue extracts of the jejunum and ileum was significantly higher in the LG2055 fed group compared with the control group (Figure 1D). In the intestinal lavage fluid, the amount of IgA in the LG2055 group was significantly higher than that in the control group, but the amount of IgG in the serum was not different for the two groups (Figure S1A, C). The amount of fecal IgA in the LG2055 fed group was significantly higher after 5 weeks than that in the control group (Figure S1B). The populations of IgA^+^ lymphocytes (IgA^+^B220^+^ cells) in PP cells and IgA^+^ plasma cells (IgA^+^B220^-^ cells) in LP cells were higher in the LG2055 group than that in the control group (Figure 1E, F). When mice were fed the control diet or the diet supplemented with LG2055 for 10 days, the amount of total IgA in the intestine of the LG2055 fed group were slightly higher in the jejunum and significantly higher in the colon compared with the control group (Figure S2), suggesting that oral administration of LG2055 for at least 10 days is required to induce the IgA production in the intestine. We also ascertained that administration of heat-treated LG2055 significantly increased the total IgA level in the jejunum extracts, but that the effect was not as strong as with the non-heat-treated LG2055 (Figure S3).

### Dendritic cells are crucial for the induction of IgA production by LG2055

**Figure 2:**
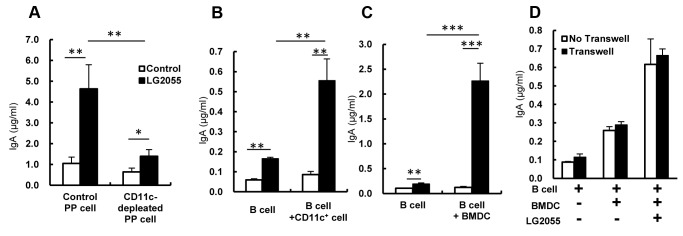
Significance of dendritic cell for the induction of IgA production by LG2055. (A) Whole PP cells or CD11c^+^ cell-depleted PP cells were cultured with or without heat treated LG2055 (10 µg/ml) for 7 days. (B) B cells from the spleen were co-cultured with or without CD11c^+^ cells derived from PP cells (left) or BMDC (right) in the presence or absence of the LG2055 for 7 days. (C) B cells were co-cultured with or without BMDC in the presence or absence of LG2055 in Transwell system for 7 days. The amounts of IgA in culture supernatants were determined by ELISA. Representative data from three for PP cells or four for BMDC independent experiments are shown. Each experiment was done with triplicate cultures; data are shown as the mean ± SD. * P<0.05, ** P<0.01, *** P<0.001 was shown by *t*-test.

To investigate the mechanisms for the induction of IgA production by LG2055, we determined and compared the amounts of total IgA in the supernatants of PP cells and PP cells depleted of CD11c^+^ cells cultured with or without LG2055 for 7 days. In the control PP cells, LG2055 markedly induced the IgA production, while in PP cells depleted of CD11c^+^ cells, the induction level of IgA by LG2055 was significantly lower than that in the control PP cells (Figure 2A). To determine the function of DC that is significant in the induction of IgA production by LG2055, B cells were cultured with or without CD11c^+^ cells isolated from PP cells or BMDC in the presence or absence of LG2055. The LG2055 increased IgA production when B cells alone were cultured, however the amount of IgA production when B cells were cultured with CD11c^+^ cells from PP cells or BMDC was more strongly increased (Figure 2B, C). Though LG2055 also increased the amount of IgG production when B cells were cultured with BMDC, the level of IgG production was very little (about 10 ng/ml) (Figure S4), compared with that of IgA (about 2 µg/ml) (Figure 2C).The induction level of IgA production by LG2055 in the B cell and BMDC co-culture system was highest among the tested four strains (Figure S3). To elucidate whether diffusible factors or interaction between B cells and BMDC was critical for the IgA production by LG2055, BMDC and B cells were cultured by a transwell arrangement where the BMDC were unable to directly contact the B cells, thereby restricting the interaction to only diffusible factors, and measured the IgA amounts generated. The LG2055-induced IgA production levels with the transwell arrangement were similar to those in the conventional culture (Figure 2D). This result suggests that diffusible factor(s) from the BMDC induced by LG2055 is/are necessary for the LG2055-induced IgA production in this culture system.

### Identification of BMDC-derived factors involved in IgA production by LG2055 stimulation

**Figure 3:**
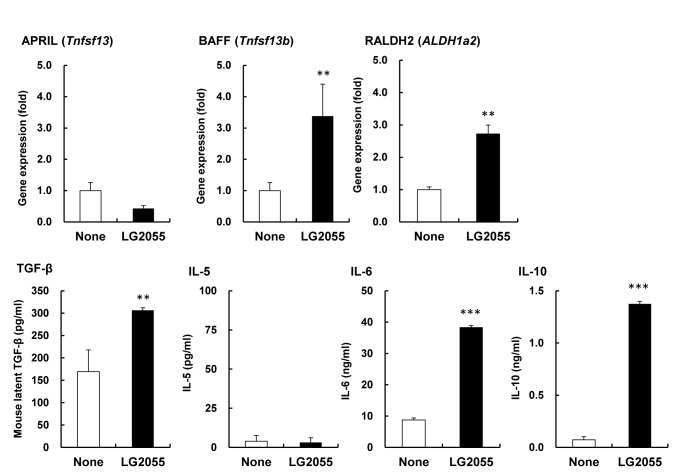
Gene expression and cytokine production of BMDC stimulated by LG2055. BMDC was cultured with or without LG2055 for 48 hours. Gene expression of APRIL (*tnfsp13*), BAFF (*tnfsp13b*), RALDH2 (*aldh1a2*) in BMDC was determined by quantitative PCR. Amounts of TGF-β, IL-5, IL-6, and IL-10 in the culture supernatants were determined by ELISA. Representative data from three independent experiments are shown. Each experiment was done with triplicate cultures; data are shown as the mean ± SD. ** P<0.01, *** P<0.001 was shown by *t*-test.

To identify diffusible factors from BMDC induced by LG2055, BMDC were stimulated with LG2055 for 48 h and the gene expression level of IgA-inducing factors in BMDC and the amount of IgA-inducing cytokines in the culture supernatants were measured. The gene expression of BAFF and RALDH2 increased significantly in the LG2055-treated cells compared with the control cells, but the expression of APRIL did not increase (Figure 3). The amounts of TGF-β, IL-6, and IL-10 increased significantly in the supernatants of LG2055-treated cells when compared with those of the control cells, but the amount of IL-5 did not increase (Figure 3). The induction level of these cytokines production from BMDC stimulated with LG2055 was significantly higher than that with LG1131T or LA2062 (Figure S4). These results suggest that there are multiple factors that are critical for IgA production and are induced in the BMDC stimulated with LG2055, and that these factors promote the secretion of IgA from B cells.

**Figure 4:**
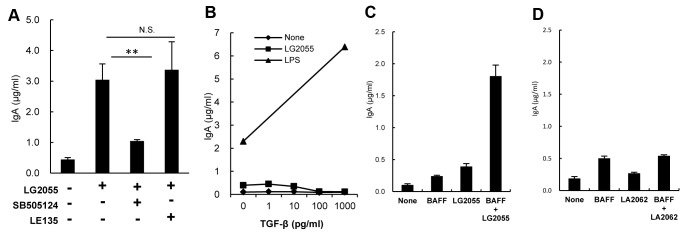
Critical factors for induction of IgA production by LG2055. (A) B cells and BMDC were co-cultured with or without LG2055 in the presence or absence of the TGF-β type I receptor inhibitor SB505124 or RAR antagonist LE135 for 7 days. (B) B cells were cultured with or without LG2055 or LPS (10 µg/ml) in the presence or absence of TGF-β for 7 days. B cells were cultured with or without LG2055 (C) or LA2062 (D), in the presence or absence of BAFF (500 ng/ml) for 7 days. IgA amounts in the supernatants were determined by ELISA. Representative data from three independent experiments are shown. Each experiment was done with triplicate cultures; data are shown as the mean ± SD. ** P<0.01, was shown by *t*-test.

To investigate the functional significance of TGF-β and RALDH2 in LG2055-induced IgA production, a TGF-β receptor I inhibitor (SB505124) or an RAR antagonist (LE135) blocking TGF-β or RA signaling was added in the B cell and BMDC co-culture. Blocking of TGF-β signaling inhibited LG2055-induced IgA production, but the RAR antagonist did not (Figure 4A), indicating that TGF-β signaling plays a critical role in LG2055-induced IgA production. As TGF-β is known as critical for IgA-class switching of naïve B cells, it was further examined whether TGF-β induced the IgA production from B cells in the presence or absence of LG2055. The TGF-β induced IgA production from B cells stimulated with LPS, as previously reported [30], [31], while with or without LG2055 stimulation it did not induce IgA production from B cells (Figure 4B). Next the effects of BAFF on IgA production in the presence or absence of LG2055 and LA2062 were evaluated. Each of BAFF and LG2055 induced IgA production, and combined stimulation with BAFF and LG2055 enhanced the induction of IgA production more markedly (Figure 4C), but combined stimulation with BAFF and LA2062 did not (Figure 4D).

### TGF-β signaling is crucial for the production of IgA-inducing factors from BMDC stimulated by LG2055

**Figure 5:**
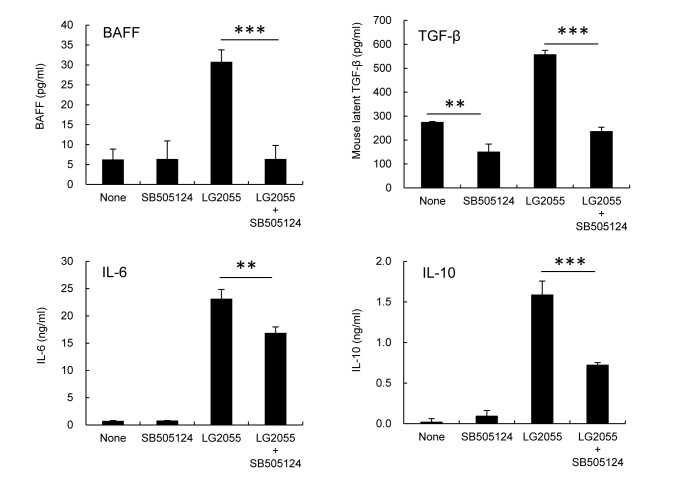
Importance of TGF-β type I signaling for the cytokine production from BMDC by LG2055. BMDC was cultured in the presence or absence of LG2055 and TGF-β type I receptor inhibitor SB505124 for 48 hours. Amounts of TGF-β, IL-6, and IL-10 in the culture supernatants were determined by ELISA. Representative data from two independent experiments are shown. Each experiment was done with triplicate cultures; data are shown as the mean ± SD. ** P<0.01, *** P<0.001 was shown by *t*-test.

We investigated whether TGF-β signaling was critical for the production of IgA-inducing factors from BMDC stimulated by LG2055. TGF-β type I receptor inhibitor (SB505124) completely inhibited BAFF and TGF-β induction and partially inhibited IL-10 and IL-6 induction in BMDC stimulated by LG2055 (Figure 5). These results demonstrate that endogenous TGF-β contributes to the production of BAFF, TGF-β, IL-10, and IL-6 from BMDC stimulated by LG2055.

### LG2055 induces IgA production via TLRs

**Figure 6:**
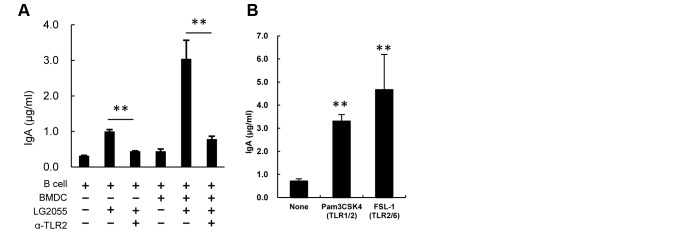
Significance of TLR2 in the induction of IgA production by LG2055. B cells or both B cells and BMDC were cultured with or without LG2055 in the presence or absence of the anti TLR2 antibody for 7 days. (B) Pam3CSK4 (TLR1/2 ligand) or FSL-1 (TLR2/6 ligand) was added to the B cell and BMDC co-culture system, and cultured for 7 days. The amounts of IgA in culture supernatants were determined by ELISA. Representative data from three independent experiments are shown. Each experiment was done with triplicate cultures; data are shown as the mean ± SD. ** P<0.01 was shown by *t*-test.

A previous report has shown that LG2055 stimulated total IgA production in splenocyte cultures from TLR4-knockout mice, but not in splenocyte cultures from TLR2-knockouts [27], and here we examined whether LG2055-induced IgA production in the B cell and BMDC co-culture was dependent on the function of TLR2. Addition of anti-TLR2 antibodies significantly inhibited LG2055-induced IgA production in both B cells alone and B cell and BMDC co-cultures (Figure 6A). Furthermore, Pam3CSK4 and FSL-1, which are ligands of TLR1/2 and TLR2/6, respectively, induced the IgA production in B cell and BMDC co-cultures (Figure 6B). These results demonstrate that LG2055 induces IgA production via TLR2, in B cells only, as well as in B cell and BMDC co-cultures.

## Discussion

*L. gasseri* is a predominant *Lactobacillus* species in the human small intestine [32] and the *L. gasseri* strain, LG2055 has been isolated from human feces. We have previously reported that LG2055-fed mouse dams reduced rotavirus (RV) infections in pups and showed elevated RV-specific IgA levels in the breast milk originating from the stomach [27]. In the present study, we demonstrate that administration of LG2055 induces IgA production in the mouse small intestine. Induction of IgA production by LG2055 is considered to prevent the invasion of harmful microorganisms and toxins through the epithelial cells in the small intestine, the initial site of adherence and infection of pathogens in the gut. There are many reports that some probiotic bacteria strains induce IgA production in the intestine. However, not all probiotic strains have the ability to induce production of IgA *in vivo*, even when the induction of IgA production is confirmed by *in vitro* studies [13], [33], [34]. It is known that different probiotic strains have different properties and that it is not possible to extrapolate the effects of one probiotic strain to others or the effect of one strain with a specific pathogen to other pathogens [35]. In this investigation it is established that LG2055 exhibits the induction of IgA production both *in vivo* and *in vitro*. Furthermore, the effect of induction of IgA production by LG2055 is clearly stronger than that of the *L. gasseri* type strain or of other *Lactobacillus* species *in vitro* (Figure 1A and S3). Thus, LG2055 might be a strain that has the high potential to induce IgA production among *Lactobacillus* strains. Recent findings have shown that secretion of IgA is critical in the regulation of the composition of the microbial community in the gut [36]–[38]. Oral administration of LG2055 in humans is able to lower both faecal bacterial population of *Staphylococcus* and faecal concentration of p-cresol. [21], [22]. Induction of IgA production by LG2055 should play an important role in the control of the intestinal microflora.

The DCs play a critical role in the induction of IgA production in the gastrointestinal tract [39], [40], and a large number of soluble factors produced by the DCs in the gut are involved in the induction of IgA production (e.g., TGF-β, IL-6, IL-10, APRIL, BAFF, and retinoic acid) [41]. It has been reported that interaction between probiotic bacteria and DCs modulates cytokine production as well as the function of DCs [42], [43]. These reports prompted us to investigate the role of DCs in the induction of IgA production by LG2055. The data here using an *in vitro* co-culture of B cells with either CD11C+ cells derived from PP cells or BMDC showed that the DCs play an important role in the augmentation of IgA production by LG2055 (Figure 2). We also observed that the gene expression and protein production of IgA-inducing mediators, BAFF, RALDH2, TGF-β, IL-6, and IL-10, were up-regulated in LG2055-stimulated BMDC (Figure 3). As being reported, TGF-β and BAFF induce an IgA class-switch recombination, and IL-6 and IL-10 induce differentiation of IgA-producing plasma cells in IgA^+^ B cells [30]. It is possible that the interaction between LG2055 and DCs may contribute to both IgA class-switching and plasma cell differentiation. This is supported by the result that oral administration of LG2055 in mice increased the rate of IgA^+^ lymphocytes in PP cells and IgA^+^ plasma cells in LP cells (Figure 1E, F). The induction level of BAFF, TGF-β, IL-6, and IL-10 production from BMDC stimulated with LG2055 was significantly higher than that stimulated with LG1131T or LA2062 which had low potential to induce the IgA production (Figure S4). The high induction of these cytokines by LG2055 may be one of the reasons for the higher induction level of IgA production by LG2055 compared with that by the other *Lactobacillus* strains.

Recently, it has been reported that DCs-derived retinoic acid promotes IgA secretion [44] and retinoic acid-treated BMDC induces IgA-secreting B cells, through promotion of TGF-β production from BMDCs [45]. Though the gene expression of RALDH2 (*ALDH1a2*), which is an enzyme responsible for the conversion of retinal into retinoic acid and the major isoform expressed in the gut-associated DCs, was up-regulated in LG2055-stimulated BMDC, LG2055 induced IgA production in the presence of the RAR antagonist, LE135 (Figure 4A). At the same time, with the TGF-β type I receptor inhibitor SB505124 there was no LG2055-induced IgA production (Figure 4A), indicating that the TGF-β signal transduction is critical for IgA production by LG2055 in the B cell-BMDC co-culture, while RAR signal transduction is not essential. Recent evidence has indicated that gut-associated DCs induce the expression of gut-homing receptors on B cells, via a mechanism that depends on retinoic acid. This fact implies the possibility that LG2055 contributes to the induction of IgA production via a promotion of the gut-homing of B cells, as the gene expression of RALDH2 was increased in the small intestine of the LG2055-administrated mice (data not shown).

Our results confirm previous observations that TGF-β induced IgA production in LPS-stimulated B cells [30], but not in LG2055-stimulated B cells (Figure 4B). It is noteworthy that the amount of IgA produced from B cells stimulated by LG2055 markedly increased in the presence of BAFF (Figure 4C). The BAFF and APRIL are shown to enhance immunoglobulin class switching to IgA in B cells [46], [47]. In addition, it is reported that TACI expression is up-regulated in B cells stimulated by oligodeoxynucleotides (CpG ODN), and the up-regulation of TACI, working as BAFF and APRIL receptors, on B cells render these to induce IgA secretion [48]. Further, it is demonstrated that the TLR ligands cooperate with the TACI ligands to induce antibody secretion [49]. The results here show that LG2055-induced IgA production was strongly inhibited by the treatment of anti TLR2 antibody both in the B cell only culture and in the B cell-BMDC co-culture (Figure 6A). From this it may be surmised that LG2055, cooperatively with BAFF stimulates B cells to induce IgA production, mainly through the TLR2 signaling pathway. Our results indicated that LG2055 solely induced IgA-production from B cells in the absence of BMDC or BAFF, but the reasons for this practice are unclear. As we also showed, LPS-stimulated B cells induce IgA-production to some extend in the absence of TGF-β, though they massively induce IgA-production in the presence of TGF-β. The induction level of IgA-production from B cells stimulated with LG2055 and/or BAFF appears to be similar to that stimulated with LPS and/or TGF-β. LPS and LG2055 may be involved in proliferation or survival of B cells, post-switched, but not yet expressed IgA. Further investigation is needed to elucidate how LPS or LG2055 solely induces IgA-production from B cells. LA2062 did not stimulate B cells with BAFF to induce IgA production (Figure 4D). The biochemical mechanism for the difference between LG2055 and LA2062 effect remains unexplained. Amounts of peptidoglycan and lipoteichoic acid present in the cell walls of *Lactobacillus* species widely differ with respect to the each strain [50]. The difference of the capability of IgA-induction between LG2055 and LA2062 may depend on the amounts of TLR2 ligand present in the each strain.

The results in Figure 4A demonstrate that LG2055-induced IgA production in B cells and the BMDC co-culture was inhibited by the TGF-β type I receptor inhibitor, even when the addition of exogenous TGF-β1 did not induce IgA production by LG2055-stimulated B cells (Figure 4B). These results suggest that the target cell of TGF-β may be BMDC, and not the B cells. The results here also show that the induction of IgA-inducing factors in LG2055-stimulated BMDC is inhibited by TGF-β type I receptor inhibitors, among which BAFF and TGF-β are completely inhibited, IL-10 and IL-6 partially (Figure 5). These results demonstrate that the TGF-β signal contributes to the production of IL-6, IL-10, BAFF, and TGF-β itself by LG2055-stimulated BMDC. It has been reported that exogenous TGF-β1 stimulates the induction of BAFF expression on mouse macrophages [51]. We confirmed that the addition of exogenous TGF-β1 enhanced the gene expression of BAFF in BMDC (data not shown). The TGF-β1 acts directly on skin-resident DCs in an autocrine/paracrine manner to develop Langerhans cells and to inhibit inflammation-induced migration [52], [53]. Further, it is revealed that autocrine TGF-β sustains the default tolerogenic function of CD8^+^ DCs [54]. Yet, no details are known of TGF-β capability in an autocrine/paracrine manner on the regulation of IgA production by DCs. Here our results imply that endogenous TGF-β from LG2055-stimulated BMDC act on BMDC itself in an autocrine/paracrine manner and contributes to the production of IgA-inducing factors, at least in this *in vitro* culture system. On the other hand, given that TGF-β1 cooperates with the CD40 ligand (CD40L) to generate antigen-specific IgA^+^ B cells in PPs [55], [56], this allows the assumption that TGF-β produced by LG2055-stimulated DCs could also act directly on B cells with CD40L and contribute to the induction of IgA production *in vivo*.

In conclusion, this study demonstrated the augmentation of IgA production in the mouse small intestine by oral administration of LG2055 and elucidated the detailed molecular mechanisms for the induction of IgA production by using a B cell and BMDC co-culture system *in vitro*. Specifically, the results suggest that LG2055 activates both DCs and B cells to induce the IgA production, and TLR2 signal is critical for its production. Further, we show that TGF-β produced by LG2055-stimulated BMDC acts on BMDC in an autocrine/paracrine manner and induces the production of IL-6, IL-10, BAFF, and TGF-β itself from BMDC to induce the subsequent IgA production (Figure S5).

## Supporting Information

Figure S1 **Effect of oral administration of LG2055 on amounts of IgA in the intestinal lavage fluid and feces, and IgG in the serum.** LG2055 was orally administrated to BALB/c mice for 5 weeks. Amounts of total IgA in intestinal lavage fluid (A), feces on 0 and 5 weeks after administration (B), and IgG in the serum (C) were determined by ELISA. Representative data from two independent experiments are shown. Data are shown as the mean ± SD (number of mice n = 10). Significant difference from control group at *P<0.05, **P<0.01 was shown by the *t*-test.
(TIF)

Figure S2 **Effect of oral administration of LG2055 for 10 days on the production of IgA in the intestine.** LG2055 was orally administrated to BALB/c mice for 10 days. Amounts of total IgA in intestinal tissue extracts were determined by ELISA. Data are shown as the mean ± SD (number of mice n = 10). Significant difference from control group at *P<0.05, **P<0.01 was shown by the *t*-test.
(TIF)

Figure S3 **Comparison of IgA induction of heat-treated LG2055 with that of non-treated LG2055 in the mouse small intestine.** Heat-treated LG2055 (heated at 80°C for 30 min) or non-treated LG2055 was orally administrated to BALB/c mice for 5 weeks. Amounts of total IgA in small intestinal tissue extracts were determined by ELISA. Data are shown as the mean ± SD (number of mice n = 10). Significant difference among groups at *P<0.05, **P<0.01 was shown by one-way ANOVA and Tukey-Kramer post test.
(TIF)

Figure S4 **Effect of LG2055 treatment on IgG production by B cell co-cultured with or without BMDC.** B cells from the spleen were co-cultured with or without BMDC in the presence or absence of the heat treated LG2055 (10 µg/ml) for 7 days. The amounts of IgG in culture supernatants were determined by ELISA. Each experiment was done with triplicate cultures; data are shown as the mean ± SD. * P<0.05 was shown by *t*-test.
(TIF)

Figure S5 **Comparison of IgA induction among four strains of** ***Lactobacillus*** **species.** Each of the four *Lactobacillus* strains (LG2055, *L. gasseri* JCM1131^t^ (LG1131T), *L. helveticus* SBT2171 (LH2171), *L. acidophilus* SBT2062 (LA2062)) was added to the B cell and BMDC co-culture system, and cultured for 7 days. The amounts of IgA in culture supernatants were determined by ELISA. Each experiment was done with tripricate cultures; data are shown as the mean ± SD. Values for stimulated cells are compared with value for non-stimulated cells by one-way ANOVA and Dunnett's post test. Significant differences are indicated by ** P<0.01.
(TIF)

Figure S6 **Comparison of cytokine production of BMDC among three strains of** ***Lactobacillus*** **species.** BMDC was cultured with or without LG2055, LG1131T, LA2062 for 48 hours. Amounts of BAFF, TGF-β, IL-6, and IL-10 in the culture supernatants were determined by ELISA. Each experiment was done with triplicate cultures; data are shown as the mean ± SD. Values not sharing a common letter are significantly different by Tukey-Kraner multiple comparison test at p<0.05.
(TIF)

Figure S7 **Schematic illustration of hypothetical model for enhancement of IgA production by LG2055.** LG2055 activates both DC and B cell. TGF-β produced by LG2055-stimulated BMDC acts on BMDC in an autocrine/paracrine manner and induces the production of IL-6, IL-10, BAFF, and TGF-β itself from BMDC to induce subsequent IgA production. TLR2 signal is critical for the induction of IgA by LG2055, at least for B cell stimulation by LG2055.
(TIF)
