# Supplementary figures and images for: Lactobacillus gasseri SBT2055 Induces TGF-β Expression in Dendritic Cells and Activates TLR2 Signal to Produce IgA in the Small Intestine
Source: PLoS One. 2014 Aug 21;9(8):e105370. doi: 10.1371/journal.pone.0105370 (PMC4140756; doi:10.1371/journal.pone.0105370)

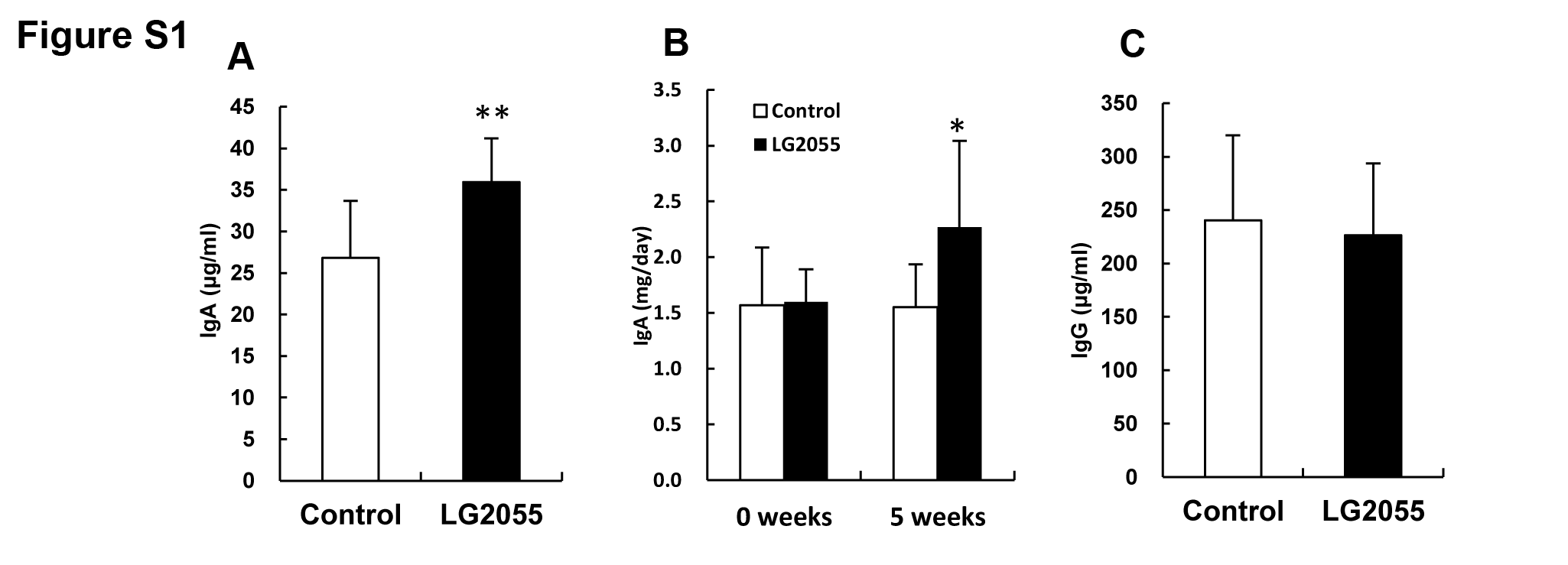

Supplement: Figure S1 — Effect of oral administration of LG2055 on amounts of IgA in the intestinal lavage fluid and feces, and IgG in the serum. LG2055 was orally administrated to BALB/c mice for 5 weeks. Amounts of total IgA in intestinal lavage fluid (A), feces on 0 and 5 weeks after administration (B), and IgG in the serum (C) were determined by ELISA. Representative data from two independent experiments are shown. Data are shown as the mean ± SD (number of mice n = 10). Significant difference from control group at *P<0.05, **P<0.01 was shown by the t-test. (TIF) [file pone.0105370.s001.tif]

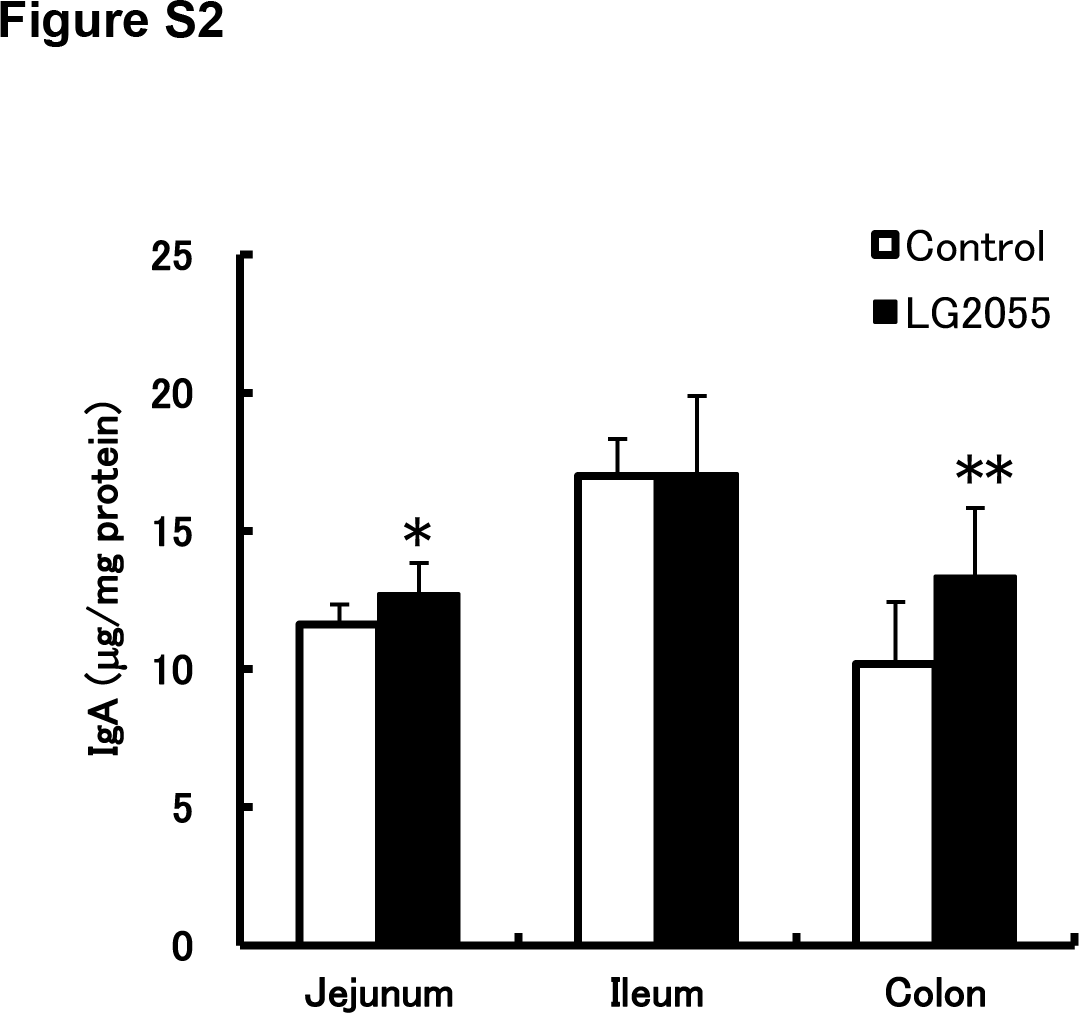

Supplement: Figure S2 — Effect of oral administration of LG2055 for 10 days on the production of IgA in the intestine. LG2055 was orally administrated to BALB/c mice for 10 days. Amounts of total IgA in intestinal tissue extracts were determined by ELISA. Data are shown as the mean ± SD (number of mice n = 10). Significant difference from control group at *P<0.05, **P<0.01 was shown by the t-test. (TIF) [file pone.0105370.s002.tif]

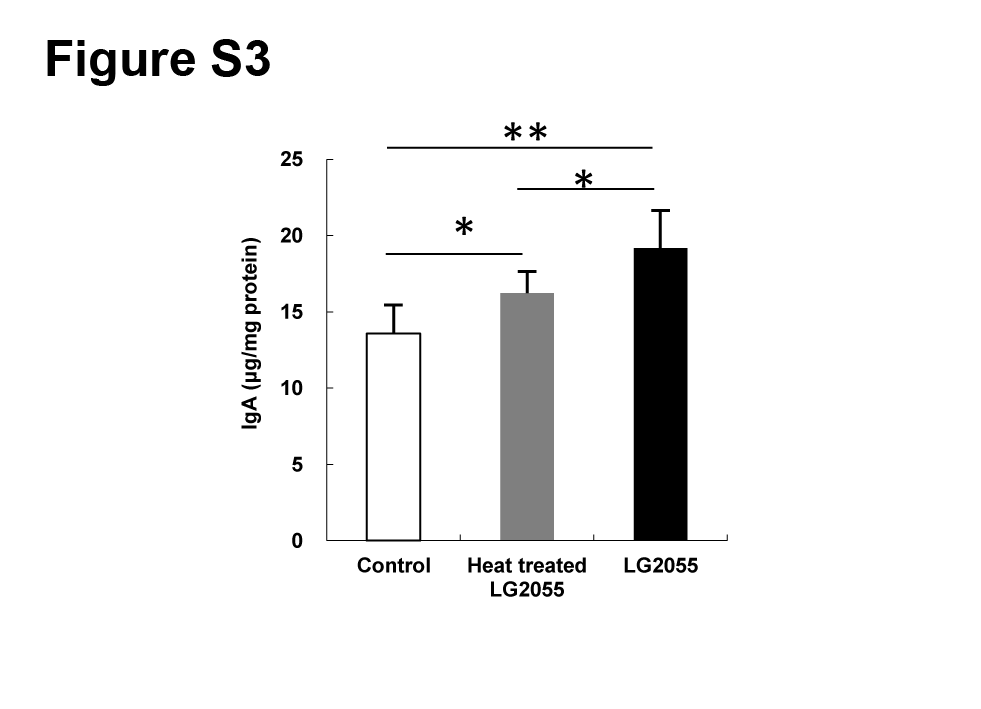

Supplement: Figure S3 — Comparison of IgA induction of heat-treated LG2055 with that of non-treated LG2055 in the mouse small intestine. Heat-treated LG2055 (heated at 80°C for 30 min) or non-treated LG2055 was orally administrated to BALB/c mice for 5 weeks. Amounts of total IgA in small intestinal tissue extracts were determined by ELISA. Data are shown as the mean ± SD (number of mice n = 10). Significant difference among groups at *P<0.05, **P<0.01 was shown by one-way ANOVA and Tukey-Kramer post test. (TIF) [file pone.0105370.s003.tif]

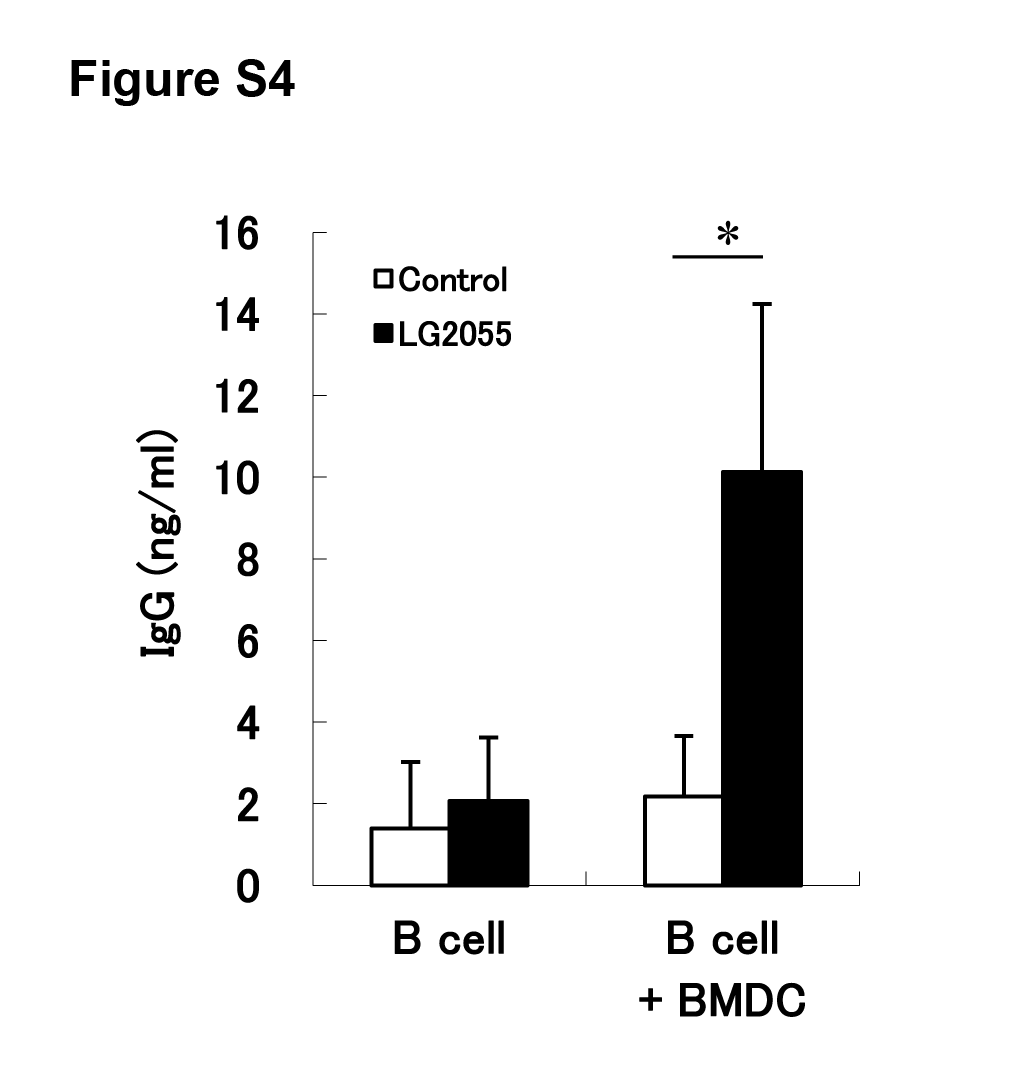

Supplement: Figure S4 — Effect of LG2055 treatment on IgG production by B cell co-cultured with or without BMDC. B cells from the spleen were co-cultured with or without BMDC in the presence or absence of the heat treated LG2055 (10 µg/ml) for 7 days. The amounts of IgG in culture supernatants were determined by ELISA. Each experiment was done with triplicate cultures; data are shown as the mean ± SD. * P<0.05 was shown by t-test. (TIF) [file pone.0105370.s004.tif]

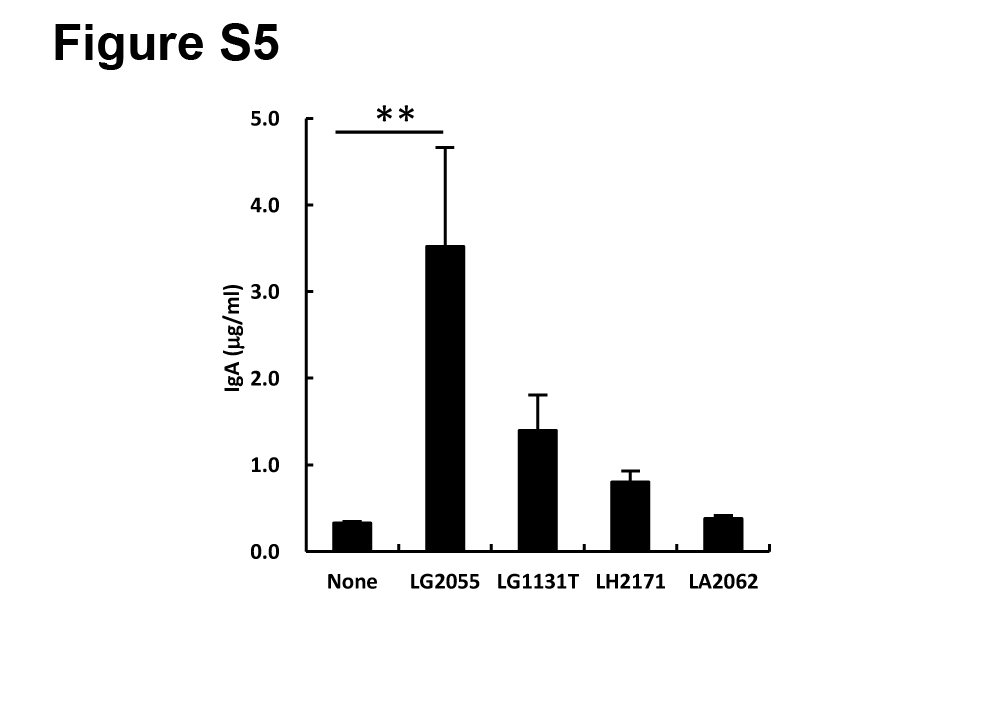

Supplement: Figure S5 — Comparison of IgA induction among four strains of Lactobacillus species. Each of the four Lactobacillus strains (LG2055, L. gasseri JCM1131t (LG1131T), L. helveticus SBT2171 (LH2171), L. acidophilus SBT2062 (LA2062)) was added to the B cell and BMDC co-culture system, and cultured for 7 days. The amounts of IgA in culture supernatants were determined by ELISA. Each experiment was done with tripricate cultures; data are shown as the mean ± SD. Values for stimulated cells are compared with value for non-stimulated cells by one-way ANOVA and Dunnett's post test. Significant differences are indicated by ** P<0.01. (TIF) [file pone.0105370.s005.tif]

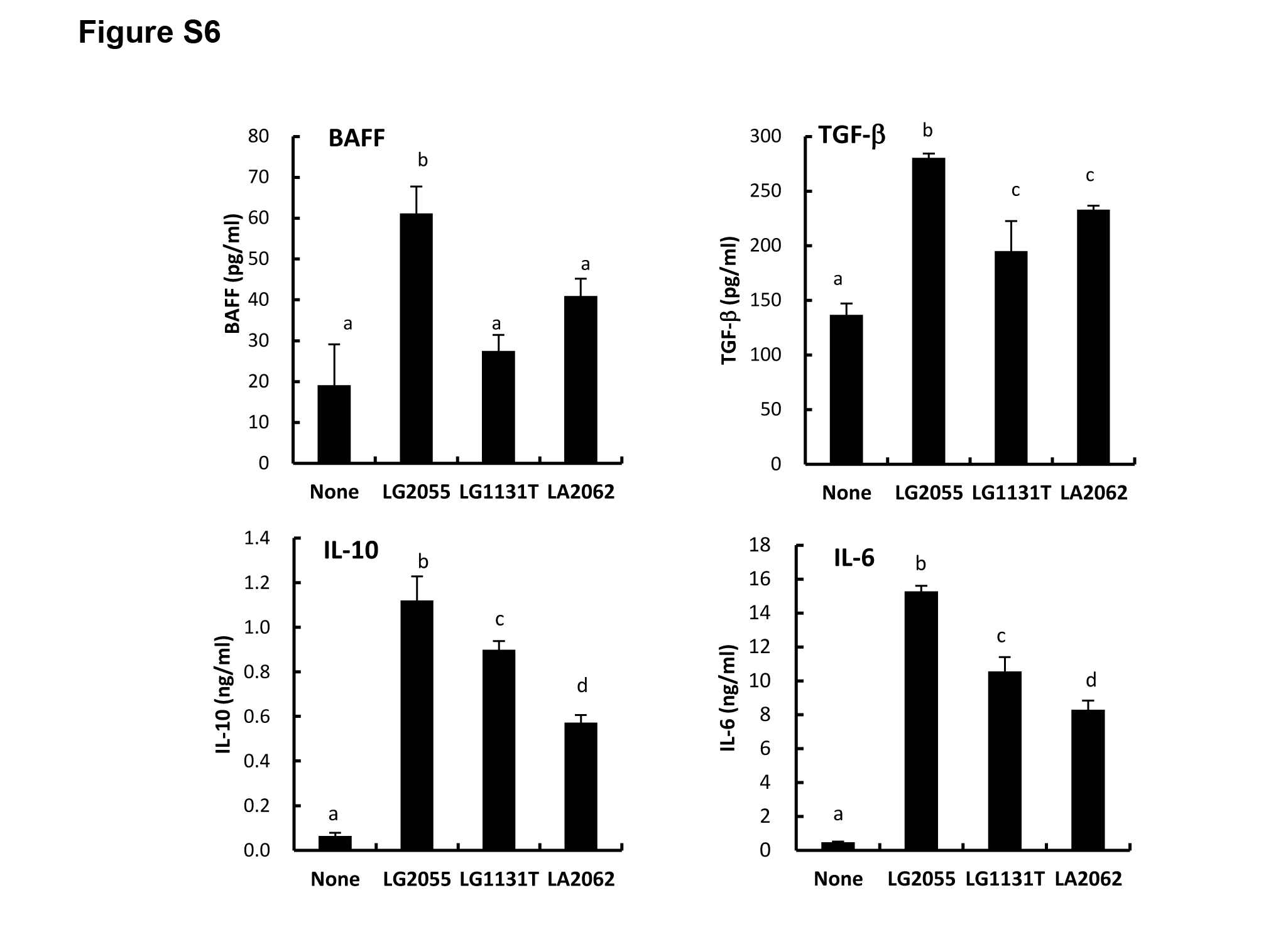

Supplement: Figure S6 — Comparison of cytokine production of BMDC among three strains of Lactobacillus species. BMDC was cultured with or without LG2055, LG1131T, LA2062 for 48 hours. Amounts of BAFF, TGF-β, IL-6, and IL-10 in the culture supernatants were determined by ELISA. Each experiment was done with triplicate cultures; data are shown as the mean ± SD. Values not sharing a common letter are significantly different by Tukey-Kraner multiple comparison test at p<0.05. (TIF) [file pone.0105370.s006.tif]

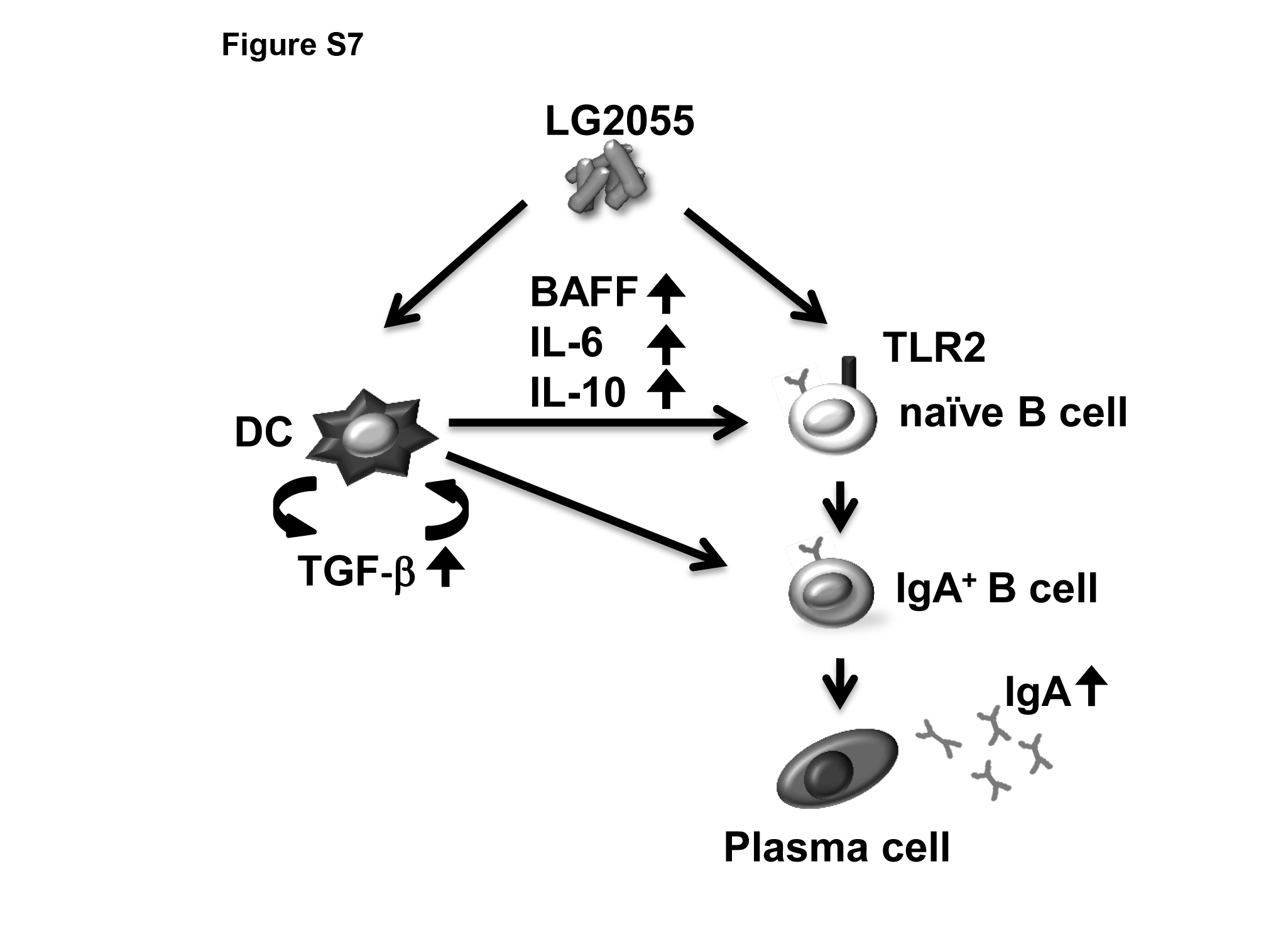

Supplement: Figure S7 — Schematic illustration of hypothetical model for enhancement of IgA production by LG2055. LG2055 activates both DC and B cell. TGF-β produced by LG2055-stimulated BMDC acts on BMDC in an autocrine/paracrine manner and induces the production of IL-6, IL-10, BAFF, and TGF-β itself from BMDC to induce subsequent IgA production. TLR2 signal is critical for the induction of IgA by LG2055, at least for B cell stimulation by LG2055. (TIF) [file pone.0105370.s007.tif]
